# Thoraco- abdominal impalement injury: a case report

**DOI:** 10.1186/1471-227X-14-7

**Published:** 2014-03-04

**Authors:** Gyanendra Malla, Bibhusan Basnet, Rais Vohra, Casey Herrforth, Shailesh Adhikari, Amit Bhandari

**Affiliations:** 1 Department of General practice and Emergency Medicine, B. P. Koirala Institute of Health Sciences, Dharan, Nepal; 2 Department of Emergency Medicine, UCSF Fresno Medical Center, Fresno, CA, USA; 3 Department of Emergency Medicine, UC Health, Emergency Medicine, CO, USA; 4 Department of Surgery, B. P. Koirala Institute of Health Sciences, Dharan, Nepal

**Keywords:** Nepal, Trauma, Emergency medicine, Trauma surgery, Rural emergency medicine

## Abstract

**Background:**

Impalement injury is an uncommon presentation in the emergency department (ED), and penetrating thoraco-abdominal injuries demand immediate life-saving measures and prompt care. Massive penetrating trauma by impalement in a pediatric case represents a particularly challenging presentation for emergency providers in non-trauma center settings.

**Case presentation:**

We report a case of 10 year old male who presented in our ED with an alleged history of fall from an approximately 15 foot tall coconut tree, landing over an upright bamboo stake approximately 50 centimeter long, resulting in a trans-abdomino, trans-thoracic injury. In addition to prompt resuscitation and hospital transfer, assessment of damage to vital structures in conjunction with surgical specialty consultation was an immediate goal.

**Conclusion:**

This article describes a case study of an impalement injury, relevant review of the available literature, and highlights the peculiar strategies required in the setting of a resource limited ED.

## Background

Thoraco-abdominal impalement is one of the most severe types of penetrating trauma, which is an under-reported yet increasing source of worldwide morbidity [1-5].

The management of impalement injuries poses specific challenges in pre-hospital care and transport. There is uniform agreement that the impaling object should be left in situ until management at a tertiary trauma center can be started [1-6]. Furthermore, targeted examination in the hospital should expedite critical, definitive treatments. We report the successful management of a complex impalement case in a rural emergency setting in Nepal. We discovered that prompt diagnostic and treatment decisions in conjunction with a collaborative trauma team leads to a favorable outcomes in non-trauma care settings.

## Case–presentation

A 10-year-old boy fell approximately fifteen feet from a coconut tree and landed on a bamboo stake. The stake penetrated the child at the left lower abdomen and exited at zone 1 of the neck resulting in nearly vertical impalement in the caudo-cephaloid direction. Bystanders uprooted the bamboo stake from the ground as gently as possible to prevent movement of the stick within the child’s abdominal and thoracic cavities as instructed by a local health worker on scene. Emergency Medical Services (EMS) personnel in Nepal are only beginning specialized training beyond basic assessment and transfer, so further interventions such fluid resuscitation was not performed on scene or enroute. Further, due to the poor internal infrastructure as a result of financial and political instability in Nepal as well as the native rugged terrain, the transport time was approximately 3 hours.

**Figure 1:**
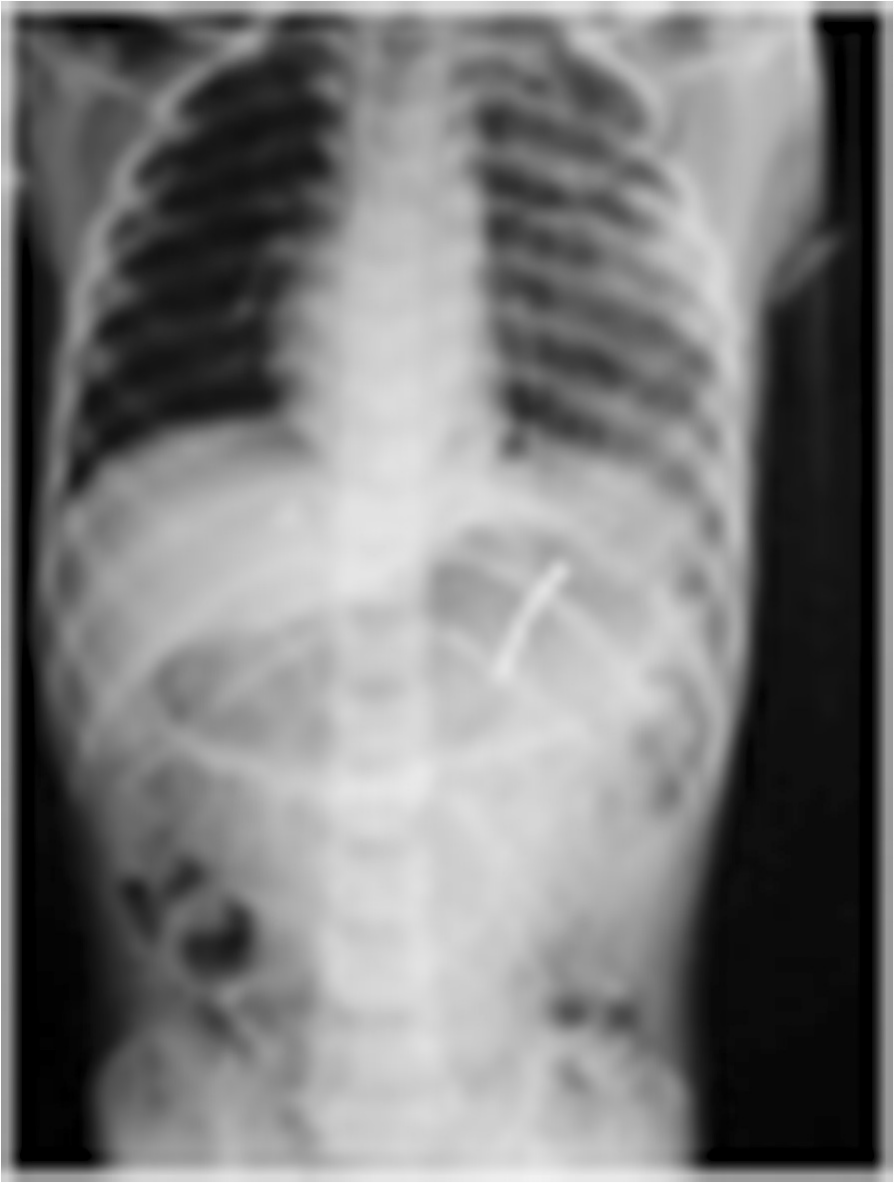
**Chest radiograph at the time of the patient’s hospital admission.** Left lung middle lobe is contused (hematoma formation), with obliteration left costo-phrenic angle. The bamboo stake is barely discernible by faint translucent lines.

**Figure 2:**
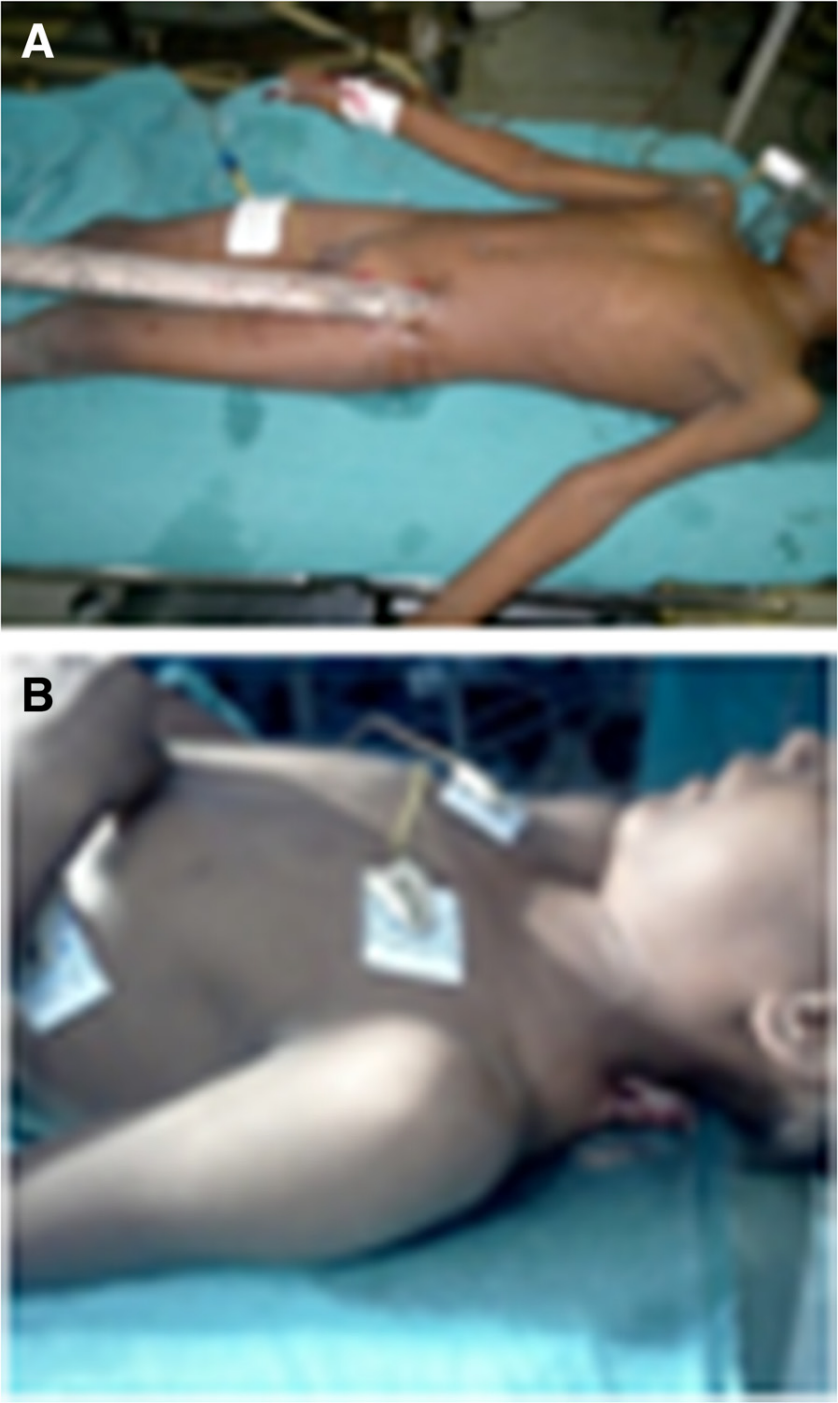
**A, B Series of photos of the Patient in the resuscitating room.** A bamboo stick impaled via the left lower abdomen exiting at zone 1 of the neck.

On presentation to the Emergency Department (ED), the patient’s airway was intact and he was fully alert (GCS 15). His breathing was labored and his initial oxygen saturation was 86% on room air. On examination, nearly absent lung sounds were auscultated in the left lung. He was placed on air mask at 5 L/min, improving oxygenation to 96%. As saturation was maintained, the surgical team deferred immediate chest thoracostomy until in the operating suite. His first blood pressure was 90/60 mmHg with a pulse of 100 BPM, so fluid resuscitation was initiated via two wide bore intravenous catheters with a bolus of 2 liters of normal saline. The abdomen was tender, with guarding and mild rigidity. A bamboo stake with an iron nail (seen in the abdominal X-ray adjoining the stomach silhouette in Figure 1) remained impaled in the body (Figure 2A and B), entering into the abdomen between the left iliac fossa and the lateral border of rectus abdominis muscle, and traversing through the whole of left side of body exiting at zone 1 of the neck. Green, foul smelling peritoneal contents were noted at the exiting end with minimal bleeding noted both at the hospital and on scene. Vaseline gauze was wrapped around the exit site to prevent air leakage/entry to the thorax. His neurological examination revealed no gross sensory or motor deficits, but due to distracting injury, cervical spine was stabilized with a cervical collar. FAST ultrasound was not done as our lack of training currently precludes its use. Urinary catheterization revealed 150 ml of clear urine.

**Figure 3:**
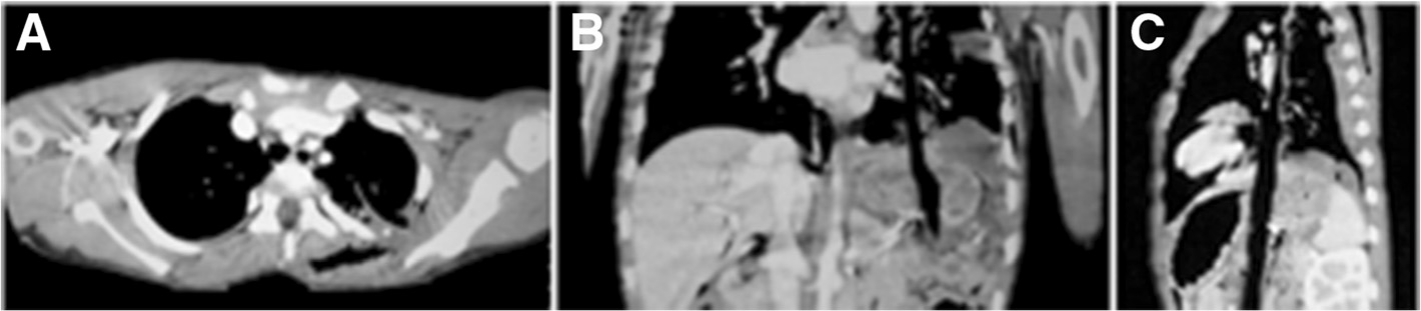
**A–C. CT scan findings on ED presentation.** The impaled piece of bamboo can be seen as a hollow air-containing tube extending vertically in the left abdomen and thorax.

The team of on-call surgeons, anesthetists and radiologists were summoned immediately. After fluid resuscitation his vitals improved (BP 118/60 mmHg, pulse of 70 BPM) and oxygenation was maintained, so we proceeded to imaging for better surgical planning. Members of the ED, surgery and anesthesia teams accompanied him to the radiology room. In concert, other members of the surgical team prepared for impending operative intervention. Antero-Posterior (AP) radiographs of the chest and abdomen were first taken (Figure 1). Again, the patient was hemodynamically stable and no haemothorax or pneumothorax was noted, so we proceeded with CT and deferred intervention such as chest thoracostomy (Figure 3A–C).

Ceftriaxone, metronidazole and tetanus vaccination were administered as per ED protocol for emergent surgeries. After the parents’ informed consent, the anesthesia team performed rapid sequence intubation using a double lumen tube for single lung ventilation in the operating theatre. The patient was placed on slight right lateral position to facilitate a left sided thoraco-abdominal surgical approach. Intra-operatively, an approximately 50-cm long bamboo stick penetrating through the anterior abdominal wall at left iliac fossa causing minimal colonic injury (AAST- OIS Grade 1), and transecting jejunum 45 cm from the duodeno-jejunal flexure (AAST- OIS Grade 5) was noted. The bamboo stake further penetrated the body of stomach and passed through the diaphragm.

In the thoracic compartment, the object had transected the left lower lobe of the lung and lacerated the upper left lobe, exiting the body from the posterior triangle of the neck. Incredibly, no major vessels were injured, and the mediastinal organs were intact, except for gross contamination with gastrointestinal contents. The bamboo stake was removed by careful dissection from the injured abdominal organs and the diaphragm as well as adequate proximal and distal vascular control.

**Figure 4:**
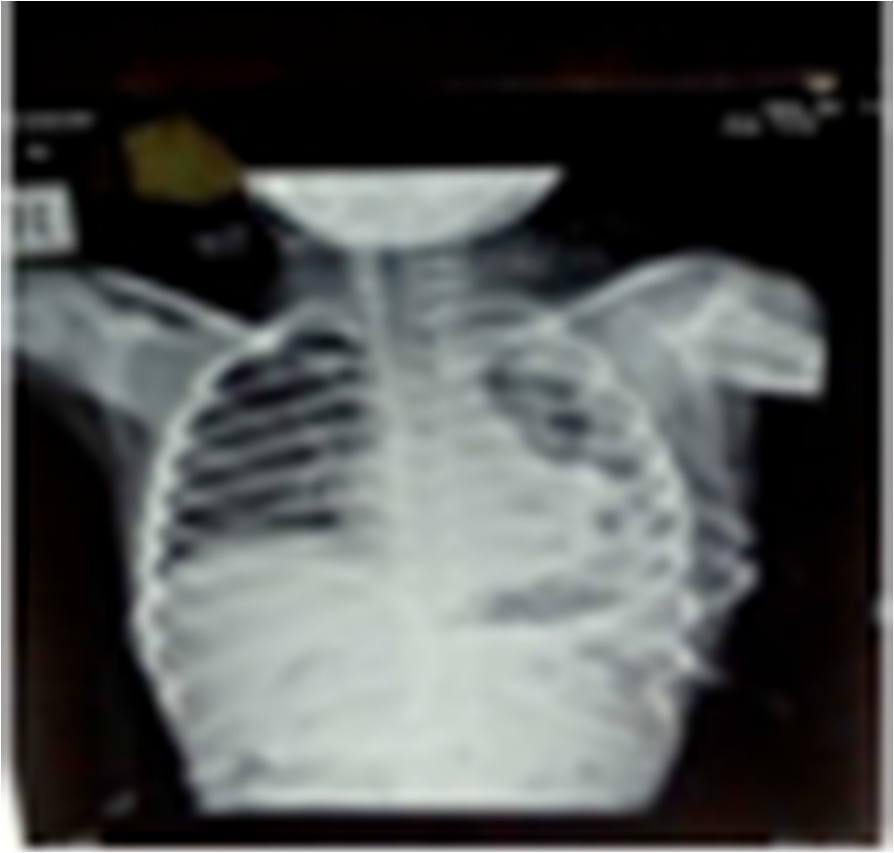
Post-operative X-Ray- showing left lower lobe lobectomy status with chest tube in situ.

A left lower lung lobectomy was done as the lower lobe was not salvageable (Figure 4), and the laceration of the upper lobe was repaired. A chest tube was inserted in 7th intercostal space. Gastric perforation was repaired in two layers (inner polyglactin and outer silk sutures). Transected jejunum was repaired with resection and end-to-end jejunal anastomosis. A thorough intra-abdominal lavage was performed with normal saline, and a left sub-hepatic drain was prepared. The intraoperative blood loss was approximately 500 ml. A brief episode of intra-operative hypotension was successfully managed with rapid infusion of crystalloids and two packs of fresh whole blood. The ED has a system of on demand fresh blood products in the hospital in case of extreme emergencies from donors within the hospital premises. After stabilization, the patient was admitted to the Intensive Care Unit (ICU).

### Post-operative management

The child remained intubated and was transferred to the ICU. Meropenem and clindamycin were added as the ICU team was concerned about contamination from organic matter and hollow viscus injury. These medications were donated free of charge. After extubation at 36 hours, he was transferred to the surgical ward. His postoperative period was complicated by superficial infection of the entry wound on the fourth hospital day, which was managed by local dressings and topical antibiotics. A psychiatric evaluation for post-traumatic stress disorder elicited no psychopathologic disorder. The child was discharged home after 21 days in the hospital and was recovering well on 1-month follow up without neurological or functional deficits.

## Discussion

Owing to the complex and rare nature of combined abdominal and thoracic impalement, no clear guidelines exist for their management especially in austere environments. Vaslef *et al.* have emphasized 3 principles of managing impalement injuries however most applicable in developed nations (See Table 1) [7]. Unfortunately, well-equipped and trained pre-hospital services are yet not organized in most resource-constrained settings such as Nepal, and there are no modern trauma centers as in developed nations.

**Table 1:** Principles of management of impalement injury

	
1)	The pre-hospital providers should leave the impaled object in situ to provide a possible tamponade effect and permit the focus on rapid transport as the goal
2)	The patient should be rapidly stabilized and transported, preferably to a trauma center and
3)	The patient should be rapidly assessed and resuscitated in the emergency department, avoiding any unnecessary tests that delay care, and then transported to the operating room for definitive care.

### Care at the scene

Medics should obtain as much information as possible about the impaled object (length, shape, material), mechanism of the injury or any potential for chemical or bacterial contamination to focus adequate first aid measures [3]. Expedient pre-hospital care can be the difference in successful resuscitations, and further medical training for our EMS personnel is an imperative for improvement of trauma care in Nepal.

### Emergency department care

A patient with an impalement injury may benefit from timely diagnostic studies to identify internal injuries, the trajectory of the impaled object, and complications of the injury needing urgent attention. Of these imaging modalities, ultrasound imaging is increasingly utilized, as it is a rapid and sensitive diagnostic tool that is available in much of the developing world. Many ED physicians have been trained in its use and utility, unfortunately this has not yet reached our ED. In our case, CT was utilized to expedite effectual surgical planning and execution. Serial clinical assessments of vital signs and mental status as well as ABGs and hematocrits can help reveal physiologic deterioration. The value of simply physically reexamining the patient serially cannot be overemphasized, especially in austere settings. These interventions can help stratify patients, as impalements with stable vital signs tend to have spared vital organs. Another intervention that may improve outcomes is administration of antibiotics. We administered ceftriaxone, metronidazole and tetanus vaccination. The decision of the ICU to further cover with meropenem and clindamycin is not supported by medical literature and reflects an area in which interdepartmental communication can improve patient care.

## Conclusion

A rare thoraco -abdominal impalement injury with damage to multiple organs was managed successfully not only because of prompt, coordinated action, but also because child was brought with foreign body in situ. Our case provides insights into how this rare injury pattern can be managed in resource-constrained settings. To summarize, the outcome after massive thoraco-abdominal impalement can be improved in rural, under-resourced settings by (a) rapid transportation with the impaled object in situ (b) targeted, succinct examination and serial reassessments in the emergency department (c) pre-operative and intraoperative antibiotic and decontamination strategies to prevent and manage infections.

### Consent

Written informed consent was obtained from the patient’s parents for publication of this case report and any accompanying images. A copy of the written consent is available for review by the Editor-In -Chief of this journal.

## Abbreviations

ED: Emergency department; ABC: Airway, breathing and circulation; CT scan: Computed tomography scan; CXR: Chest X-ray; PTC: Primary trauma care; ATLS: Advanced trauma life support; BPM: Beats per minute; EMS: Emergency medical services.

## Competing interests

The authors declare that they have no competing interests.

## Authors’ contributions

GM, SA conducted and coordinated the case. GM, BB, RV, CH and AB conceived the case report, and participated in its design. BB, AB and RV and CH drafted the manuscript and sequence alignment of the report. BB, RV and CH reviewed the literature. All authors read and approved the final manuscript.

## Pre-publication history

The pre-publication history for this paper can be accessed here:

http://www.biomedcentral.com/1471-227X/14/7/prepub
